# Effective Coordination and Governance of PGRFA Conservation and Use at the National Level—The Example of Germany

**DOI:** 10.3390/plants10091869

**Published:** 2021-09-09

**Authors:** Frank Begemann, Imke Thormann, Sarah Sensen, Karina Klein

**Affiliations:** Federal Office for Agriculture and Food, 53179 Bonn, Germany; imke.thormann@ble.de (I.T.); sarah.sensen@ble.de (S.S.); karina.klein@ble.de (K.K.)

**Keywords:** plant genetic resources for food and agriculture, conservation and use, national coordination and governance structure, Germany

## Abstract

Recognition of the importance of plant genetic resources started in Germany at the end of the 19th century. Plant research and breeding began to develop in the 1920s. Formal structures of public institutions were founded, long-term conservation facilities were established, private breeding initiatives developed. In 1990, the German reunification required an assessment of the existing research and breeding landscape. This milestone allowed a comprehensive overview of the great number of stakeholders, active in the entire range of tasks related to plant genetic resources. The Federal Ministry of Agriculture then developed a conceptual approach for an efficient governance structure and published its concept of a national programme for the conservation and sustainable use of genetic resources for food and agriculture in 2000. It recognized the sharing of decentral responsibilities among the respective public and private actors and governmental levels with dis-tributed mandates and funding. It also led to the establishment of a central information and coordination center for genetic resources, which facilitates the data sharing, communication, and co-operation among stakeholders, supports public awareness and advises the Federal Ministry on national policies and efficient European and global cooperation. It also supports efficient contributions of German stakeholders into European structures and international bodies. An equivalent conceptual approach and governance structure is recommended to be established at European level.

## 1. Historic Background until 1945

In Germany, traditional, locally adapted landraces were used in agricultural production and by the mid of the 19th century a few individuals began to select and improve such landraces for better yields. Christian Adolf Leberecht Steiger in Leutewitz (fodder beets 1825), Wilhelm von Borries in Eckendorf (fodder beets 1846), Wilfried Paulsen (potato 1846), Mathias Rabbethge, and others (sugar beets 1862) and Wilhelm Rimpau (rye 1867) were a few of them [1]. The introduction of the “squarehead” wheat from England and its appreciated value for crop production created an inspiring environment and further incentives for early breeding initiatives in Germany. In 1869, the first public quality control station for agricultural, forestry, and horticultural seed was founded at the Royal Academy for Foresters and Farmers in Tharandt. The first director, Friedrich Nobbe, published a “handbook of seed science” in 1876, which became influential for further seed research. Seed testing began and the publication of the test results created a growing demand for improved varieties by farmers and also promoted the further development of breeding companies [1].

The importance of landraces as “plant genetic resources” has been recognized in Germany as early as by the end of the 19th century, when Emanuel Ritter von Proskowetz and Franz Schindler acknowledged the “use value” of traditional landraces for agricultural production in general and plant breeding in particular at the International Agricultural and Forestry Congress in Vienna in 1890 [2].

The Russian scientist Nikolai Ivanovich Vavilov started plant explorations in 1916 and collected landraces worldwide [3]. He described centers of diversity of cultivated plants and presented them in 1927 at the 5th International Genetics Congress in Berlin. Vavilov’s work greatly influenced further activities in Germany. At the beginning of the 1930s, the General Assembly of the International Plant Breeders Association in Berlin concluded far-reaching recommendations related to the conservation of landraces of cultivated plants. Representatives of all countries were requested to approach their governments to collect and conserve the diversity of traditional landraces existing in their respective countries. For this purpose, in Germany it was recommended that appropriate farmers or institutes maintain and manage traditional landraces in the original planting areas following the old cropping practices [4].

A new Kaiser-Wilhelm-Institute of Research on Plant Breeding was founded in Müncheberg in 1927, with Erwin Baur (1875–1933) becoming its first director. Although this institute collected germplasm samples in Turkey and some Latin American countries its main concern had not been the long-term aspect of conservation but rather topics such as crop plant evolution and genetics. The institute and Erwin Baur developed many fundamental impulses for further research related to plant genetic resources. Hence, in 1943, another Kaiser-Wilhelm-Institute of Crop Plant Research was founded at the Tuttenhof farm near Vienna with its director Hans Stubbe (1902–1989), a student of Erwin Baur. Stubbe laid the foundation of the institute’s future crop plant-related research in a wider interpretation, including the collecting and conservation of global crop diversity and their research in the important botanic disciplines such as systematics, ecology, genetics, biochemistry, biophysics, and physiology [2]. By the end of World War II, the institute’s important plant collection was moved to Gatersleben in the Eastern part of Germany [5]. 

It should be noted that with the increased industrialization of German agriculture at the beginning of the 20th century, the appreciation of traditional German landrace diversity was turned upside down. While important plant explorations were organized into other countries and continents (e.g., Asia, Latin America), the NAZI regime conducted a “plant variety cleaning”. This cleaning process was meant to protect the German farmers from inferior seed and resulted in a loss of some three quarters of the varieties of the main crops from the seed market [6].

## 2. Period of a Divided Germany (1945–1990)

After World War II, Germany was divided into a Western part, the Federal Republic of Germany, and an Eastern part, the German Democratic Republic. Due to the geographical division and the different political systems of the two German parts, the plant genetic resources activities developed separately throughout the various locations. 

In Eastern Germany important crop collections were located in Gatersleben and Halle and a fruit collection existed in Dresden-Pillnitz. The collection of the Institute for Plant Breeding in Halle was transferred to Gatersleben between 1945 and 1950 [2]. At the same time, in Western Germany, a part of the collections of the former Kaiser-Wilhelm-Institute of Research on Plant Breeding was moved to the Max-Planck-Institute for Plant Breeding Research at Cologne-Vogelsang. Today’s Julius Kühn-Institute for Grapevine Breeding near Siebeldingen in the South-Western part of Germany became the location for the national grapevine collection [5]. Beside these public research institutes, many private breeding companies maintained their own breeding collections. 

### 2.1. Eastern Germany

In Eastern Germany, a plant breeding and seed service infrastructure was established by the Sowjet military administration already in 1946. While throughout the country 63 seed selection and development farms (seed stations) with a total area of 27,729 ha were set up, a central coordination mandate of these farms was taken over by the German Seed Association (DSG). The DSG was responsible for the coordination, documentation, collection, and distribution of seed and planting material. Seed production plans became obligatory. Already existing small seed initiatives and institutes could still remain independent but had to follow the instructions of the DSG [7].

In addition, crop-oriented breeding research institutes were established or continued such as the Institute Quedlinburg (vegetables), Institute Bernburg (cereals, maize, forages, special crops), Institute Groß Lüsewitz (potatoes), Institute of Crop Plant Research, Gatersleben, Institute of Phytopathology, Aschersleben, Agricultural Faculty, Martin-Luther-University Halle-Wittenberg and the Institute of Breeding Research Müncheberg. Important breeders also continued their breeding activities in Hadmersleben, Gülzow, and Klein Wanzleben.

After the foundation of the German Democratic Republic in 1949, the new German Academy for Agricultural Sciences (DAL) was founded in 1951 with Hans Stubbe becoming the first president. The former DSG breeding research institutes were now integrated in the Academy as DAL-institutes [7].

- Institute Quedlinburg (breeding research, vegetables)
- Institute Bernburg (cereals, maize) and Research Station Hadmersleben (cereals, lupines)
- Institute Klein Wanzleben (beets)
- Institute Groß Lüsewitz (potatoes)
- Institute Gülzow (cereals etc.)
- Institute of Fruit Research Dresden-Pillnitz
- Institute of Forages Paulinenaue

Essential research and breeding partners of these DAL-Institutes (later called AdL-Institutes) were the Institute of Crop Plant Research (Genebank) Gatersleben, the Institute of Phytopathology Aschersleben, the Agricultural Faculty, Martin-Luther-University Halle-Wittenberg, and numerous existing seed stations. Additional nationally owned seed and planting material companies (VVB) were responsible for country-wide seed supply. The “VVB Seed and Planting Material” comprised 110 farms with over 100,000 ha farmland area [7].

The Institute of Crop Plant Research in Gatersleben played a unique role as it was the home of the important genebank collection of plant genetic resources. The institute was placed within the German Academy for Sciences (DAW) in 1948 and, later in 1972, within the scope of the Academy of Sciences (AdW). From 1970 to 1991 this Institute was called Central Institute of Genetics and Crop Plant Research (ZIGuK) before it was renamed in 1992 in Institute of Plant Genetics and Crop Plant Research (IPK). The institute, with its first director Hans Stubbe, conducted numerous collecting and exploration missions and received plant genetic resources from other collection holders. This growing genebank collection of global importance (see Table 1) was and still continues to be intensively used for research on taxonomy, genetics, phylogeny, evolution, and breeding of cultivated plants. The genebank places emphasis and conducts research on the systematics, characterization, evaluation, and documentation of the conserved plant genetic resources as a fundamental service for the research and breeding activities.

After 1945, numerous breeding or seed companies left the Eastern part of Germany and tried to settle in the Western part of Germany. As an indication, a list of companies provided by Röbbelen [8] gives an idea of this process. Out of 74 listed companies from Eastern Germany, 40 companies lost their private identity due to the land reform and 34 companies were reported to have moved to the West. The number of companies, including the ones existing already in the Western part of Germany, with at least one variety in the official seed list, was reported to be 277 in 1949 and 264 in 1975. Many of the companies were constituted as farmers’ cooperatives or other forms of production associations [8]. 

### 2.2. Western Germany

While in Eastern Germany the research, breeding, and seed systems were almost entirely based upon public institutions and infrastructure, in Western Germany the systems were continued and built upon private initiatives and companies. In the West, public institutions were active especially in the areas of research, education, and seed quality control. The Western part of Germany was controlled after the Second World War by the USA, France, and Great Britain, leading in 1949 to a constitution as a federal republic of states (*Laender*) with different mandates at the Federal and *Laender* levels. As there was no central coordination yet, the breeder associations were also divided in three parts, the Association of Plant Breeders (VdP) in Hanover, the Bavarian Breeders’ Association (VBP) in Munich, and the South-Western Breeders’ Association (VSWP) in Stuttgart. In 1962 (VdP and VBP) and 1966 (VSWP) these three associations were merged and formed the new German Plant Breeders’ Association (BDP) in Bonn (1970). Throughout the years, many private breeding companies extended their businesses based on a particular crop focus, diversification across different crops, and/or by international cooperation [8]. 

In Western Germany, public research and education related to plant breeding and plant genetic resources were embedded in leading university institutes, especially at Stuttgart-Hohenheim, Göttingen, Freising-Weihenstephan, Hanover, Giessen, Bonn and Kiel, as well as in regional research institutions. Since the 1950s, the Max-Planck-Institute for Plant Breeding Research at Cologne-Vogelsang also worked on forages and other crops.

In 1965, the possibly unique German Federation for the Promotion of Plant Breeding (GFP) was founded in Hanover to maintain and support private breeding initiatives, to assist in knowledge transfer from latest scientific developments, to facilitate the adoption for technical implementation, to support and enable high economic value-addition and to support international cooperation [9]. As such, the GFP (today GFPi) created a link between public research and private plant breeding.

The growing necessity to also conserve plant genetic resources for future research and breeding in Western Germany was addressed and promoted especially by Hermann Kuckuck, Dieter Bommer, and the GFP. Based on their initiative, in 1970, a new genebank in Western Germany was established by the Federal Ministry of Agriculture at the Institute of Agronomy and Plant Breeding of the Federal Research Centre for Agriculture (FAL) in Braunschweig [4]. The genebank in Braunschweig was developed to provide services especially to the privately structured plant breeding system. This concept was, however, different to the much wider comprehensive plant genetic resources approach of the genebank in Gatersleben.

### 2.3. West German Recognition of International Developments

During the 1970s, the Federal Republic of Germany commenced to support international activities to promote the conservation and use of plant genetic resources. In 1971, the Consultative Group on International Agricultural Research (CGIAR) was established. A number of the international agricultural research centers of the CGIAR focused on the improvement of crop plants, collected plant genetic resources, and established genebanks to support their improvement programmes. 

The Food and Agriculture Organization of the United Nations (FAO) played a key role to support international cooperation and communication related to plant genetic resources activities. In 1974, largely initiated by the FAO, the International Board for Plant Genetic Resources (IBPGR) was created to support the research, collecting, conservation, documentation, evaluation, and use of the genetic diversity of cultivated plants worldwide. It was also active to organize a global network of genebanks holding base collections within and outside the CGIAR [10].

The growing awareness of the importance of plant genetic resources led to an international agreement at the FAO in 1983, the so-called International Undertaking on Plant Genetic Resources [11] and the establishment of a Commission on Plant Genetic Resources [12], which the Federal Ministry of Agriculture was committed to. A central element of the International Undertaking was stated in article 7.1 (a):

*“there develops an internationally coordinated network of national, regional, and international centers, including an international network of base collections in gene banks, under the auspices or the jurisdiction of FAO, that have assumed the responsibility to hold, for the benefit of the international community and on the principle of unrestricted exchange, base or active collections of the plant genetic resources of particular plant species”.*

Inspired by these formal international processes and developments in plant research, the Federal Ministry of Agriculture initiated an assessment of related activities in 1985 [10].

## 3. German Reunification (1990)—An Assessment of the Plant Genetic Resources System

While the initiation of the assessment of PGR in Western Germany was started in 1985, the results were only published in 1990 [10]. It was based on the definition of plant genetic resources as laid down in the International Undertaking [12], where plant genetic resources meant the reproductive or vegetative propagating material of the following categories of plants:

1. Cultivated varieties (cultivars) in current use and newly developed varieties;
2. Obsolete cultivars;
3. Primitive cultivars (landraces);
4. Wild and weedy species, near relatives of cultivated varieties;
5. Special genetic stocks (including elite and current breeders’ lines and mutants).

Hence, in a broad interpretation, the assessment addressed resources in Western Germany, which were [10]:

- Important for breeding of actual and potential crops as well as important from an ecology perspective and for the conservation of the vegetation in Germany;
- Required for plant breeding and land improvement, considering the scope of agriculture, horticulture, pomiculture, forestry and landscape management;
- Important in relation to nature conservation, ecosystems and protection of wild species;
- Maintained by Federal and *Laender* institutions and non-governmental organizations.

The most important institution in Western Germany at the time of reunification was the Institute of Agronomy and Plant Breeding of the Federal Research Centre for Agriculture (FAL) in Braunschweig, holding a large genebank collection. Since 1980, endangered wild species in Germany were also integrated in this collection. Reference samples of the Federal Plant Variety Office (BSA) and related information were handed over to the FAL-genebank for varieties after the expiry of their variety protection period. Evaluation and documentation of the resources were a particular priority of the institute. Annually, 7000–8000 samples were provided to recipients at their requests; about 1/3 of the requests were received from abroad [10].

Apart from the registration of test collections maintained at the Federal Plant Variety Office for the testing period, important collections of agricultural crops were held at *Laender* institutes, universities, Max-Planck institutes, and private breeding companies.

For horticultural crops, many fruit and vegetable collections were identified at the FAL, the BSA, the Federal Research Institute for Horticultural Plant Breeding in Ahrensburg and more than 20 *Laender* and county institutes as well as numerous private companies. This diverse situation was similar for genetic resources of ornamental plants, where botanic gardens, universities, and outdoor museums played a significant role as well. The Federal Institute of Grapevine Breeding in Siebeldingen and six *Laender* institutes maintained the main genetic resources collections for grapevine research and breeding.

The plant genetic resources concept with its assessment [10] also covered forest genetic resources. The Federal Forestry and Wood Research Institute, *Laender* forestry research institutes, forest administration, arboreta and private forest owner were the key actors involved. These activities were coordinated by the Federal—*Laender* Working Group “Conservation of Forest Genetic Resources” [13].

Activities for the conservation of genetic diversity of wild species were part of the nature and landscape protection measures. Federal and *Laender* research institutes, universities, botanic gardens, outdoor museums, nature conservation associations and individuals are engaged in these activities. The main conservation responsibilities, however, were located at the *Laender* level.

The main collection of microbial genetic resources existed at the German Collection of Microorganisms (DSM; today DSMZ) in Braunschweig. The DSM was also the International Depositary Authority under the Budapest Treaty on the International Recognition of the Deposit of Microorganisms for the Purposes of Patent Procedure in Germany. The collection consisted of some 4200 strains of microorganisms [10].

At the time of the German reunification in 1990, the study by Bommer and Beese [10] presented the activities related to plant genetic resources in Western Germany. However, due to the reunification an overall assessment in both parts of Germany was needed. Hence, two additional studies were conducted and are important to mention: the so-called “genebank study” issued by the Federal Ministry for Research and Technology and presented by the Association of Academic Societies of Agricultural, Forestry, Food, Veterinary and Environmental Research (DAF) [14], and the assessment by Begemann and Hammer, published by the Federal Ministry for Food, Agriculture and Forestry (BMELF) [5].

These studies provided a comprehensive picture and revealed a large number of public institutions being involved in a wide range of activities related to plant genetic resources for research and breeding. The two large genebanks at the IPK in Gatersleben and at the FAL in Braunschweig played a leading role. They also maintained long-term base collections within the international network of base collections supported by the IBPGR [15]. In addition, in 1992, the Federal Centre for Breeding Research of Cultivated Plants (BAZ) was established in Quedlinburg. The BAZ integrated the former Institute of Phytopathology in Aschersleben, the Institute of Breeding Research in Quedlinburg, the Institute of Potato Research in Groß Lüsewitz, the Institute of Fruit Research in Dresden-Pillnitz (former AdL-institutes), and the former independent Federal Institute of Grapevine Breeding in Siebeldingen and the Federal Research Institute for Horticultural Plant Breeding in Ahrensburg. An overview of the most important genebank collections at IPK, FAL and BAZ in 1992 are shown in Table 2.

Furthermore, about 100 private breeding and seed companies were operational for the German and, partially, for the international seed markets. A number of non-governmental organizations were engaged in plant genetic resources activities such as on-farm conservation and management. Pioneering examples were the Association for the Conservation of Crop Plant Diversity (VEN) founded in 1986, the Association for the Promotion of Seed Research in Biodynamic Agriculture in 1988, the Association of Pomologists in 1991 or later the Association for the Conservation and Recultivation of Cultivated Plants (VERN) in 1996.

It was evident that there were different levels of governmental mandates at the Federal and *Laender* levels. The main responsibility for nature protection, including the conservation of genetic resources, as well as for academic institutions such as universities rests with the *Laender* governments. The Federal government oversees collaborative activities across the *Laender*, providing a national policy framework, national documentation and monitoring, and providing for the international cooperation. 

The plant genetic resources system was marked by a rich but scattered research landscape, by a few multi-crop and many crop-oriented genebanks and research institutes. A certain degree of duplication of the respective public research centers, in particular between the two large multi-crop genebanks in Gatersleben and Braunschweig, was highlighted. It was recommended to integrate the collection of the genebank in Braunschweig into the genebank in Gatersleben, which was implemented over a certain period of time to avoid any loss of material or knowledge related to the collection and was concluded in 2004.

## 4. Overall Coordination and Governance Structure of Genetic Resources for Food and Agriculture

As part of the initial assessment in Western Germany, before the reunification, a plant genetic resources concept was elaborated on how to integrate the multitude of stakeholders, measures, and programmes and better prepare the entire plant genetic resources system for future challenges and opportunities [10].

Background to this so-called “Bommer and Beese” concept [10] was the recognition of the loss of species diversity and genetic erosion on the one hand and, on the other hand, new opportunities arising from recent scientific developments especially in the biological sciences and information technology, which allowed new developments of the potential use of genetic resources.

The proposed plant genetic resources concept covered a wide range of agricultural crops including fruit crops, vegetables, ornamentals, grapevine, forest, and wild species including crop wild relatives. Moreover, microorganisms were considered. It emphasized the promotion of research and conservation efforts and recognized different mandates related to the federal structure of Germany. Core elements of the concept were the following: the Federal Ministry of Agriculture itself, an inter-disciplinary Advisory Board for Plant Genetic Resources, an Information and Coordination Centre for Genetic Resources with a central documentation system, and crop committees for different crops (crop groups). Besides these new bodies, the existing system with the well-functioning conservation, research, and breeding institutions at Federal and *Laender* levels should remain as it was operating by that time [10].

This decentral or distributed system with a central coordination unit was considered advantageous over a combined and centralized system in one large plant genetic resources institution or a completely decentral system of individual institutions. Advantages were seen in securing the necessary continuity of this long-term task, in supporting the inter-disciplinary collaboration, in maintaining the plant genetic resources activities as part of a broader institutional research setting, and in using available infrastructure for new conservation measures [10].

A first step to implement the new components of the plant genetic resources concept was the establishment of the proposed Information and Coordination Centre for Genetic Resources (IGR) in April 1991. The IGR was located at the German Centre for Agricultural Documentation and Information (ZADI), an institution under the Federal Ministry of Agriculture. The IGR started and evolved in a stepwise manner. Initially, the main task was to develop a national database for plant genetic resources and support the exchange of data with other national and international databases. As from 1993, with its new director Frank Begemann, the following tasks were added:

- To provide advice to the Federal and *Laender* ministries;
- To support the Federal Ministry of Agriculture
  ○ To represent Germany in international bodies such as the FAO Commission on Plant Genetic Resources, the European Cooperative Programme for Plant Genetic Resources (ECPGR) and bodies at the European Commission;
  ○ To prepare the 4th International Technical Conference of FAO for Plant Genetic Resources (1996) in Leipzig;
- To provide the Secretariat to the National Committee that was asked to prepare the German national report for this 4th International Technical Conference of FAO;
- To collect, analyze and disseminate information about national and international conservation measures;
- To undertake public awareness activities;
- To support collaboration between the formal and informal sectors;
- To support national conservation measures in line with international activities;
- To contribute to improved links between conservation and use activities.

The National Committee that was created for the 4th International Conference of the FAO, consisted of representatives of all stakeholders involved such as different ministries, science and research, the private sector, non-governmental organizations, international cooperation agencies, associations, farmers organizations, and nature conservation agencies [16]. This inclusive composition proved to be very useful and was taken up a few years later when a formal national committee for plant genetic resources had to be formed.

It is worth mentioning that the IGR could not support and coordinate activities through a dedicated budget line under its control. It rather facilitated conservation, documentation and sustainable use of plant genetic resources merely through appropriate information and communication means. This turned out to be effective and supported the collaboration between stakeholders within the large and diverse plant genetic resources system in Germany. 

Step by step, the usefulness of these information and coordination functions of the IGR in the plant domain was recognized by the Federal Ministry officials in the domains of animal, forest, and aquatic genetic resources.

Based on this experience, and considering international processes such as the broadening of the scope of the FAO Commission on Plant Genetic Resources to the new Commission on Genetic Resources for Food and Agriculture (CGRFA) in 1996, the German Federal Ministry of Food, Agriculture and Forestry published a landmark “Concept for the Conservation and Sustainable Use of Genetic Resources for Food, Agriculture and Forestry” in 2000 [17]. This concept was built on the former West German concept for plant genetic resources [10].

**Figure 1:**
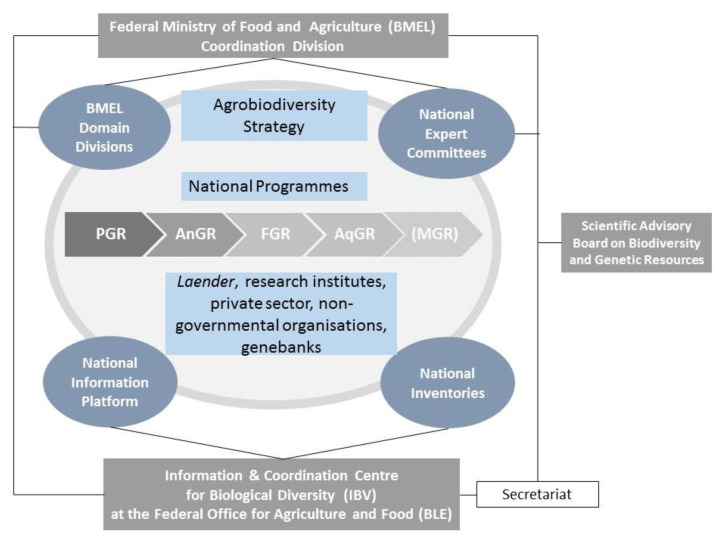
Coordination and governance structure of the National Concept for the Conservation and Sustainable Use of Genetic Resources for Food, Agriculture and Forestry (revised from [17]).

As far as the coordination and governance structure is concerned, in essence, the genetic resources concept of 2000 [17] is still operational today. The main components of this coordination and governance structure with updated names of their functional entities are (Figure 1):

- Federal Ministry of Food and Agriculture (BMEL) with a coordination division related to genetic resources for food and agriculture and additional domain-specific divisions for plant, animal, forest and aquatic genetic resources;
- Agrobiodiversity strategy and national programmes for plant, animal, forest and aquatic genetic resources;
- National expert committees for plant, animal, forest and aquatic genetic resources, consisting of *Laender* authorities and a wide range of experts and stakeholders.
- Scientific Advisory Board for Biodiversity and Genetic Resources;
- Information and Coordination Centre for Biological Diversity (IBV) (successor of the former IGR) at the Federal Office for Agriculture and Food (BLE);
- National inventories for plant, animal, forest and aquatic genetic resources;
- National information platform/website (https://genres.de/en/)

In 2007, the Federal Ministry of Food, Agriculture and Consumer Protection (BMELV) published a so-called “Agrobiodiversity Strategy”, in furtherance of the genetic resources concept from 2000 and as a supplement to the National Biodiversity Strategy [18]. The title of the Agrobiodiversity Strategy also sums up the mission statement: “Preserving agrobiodiversity, tapping the potential of agriculture, forestry and fisheries and making sustainable use of it”. To achieve this, the strategy has three overarching aims:

- Achieve long-term conservation and broader-based use of genetic resources;
- Achieve sustainable use of agricultural biodiversity while protecting natural ecosystems and threatened species;
- Strengthen international cooperation and a globally coordinated strategy for the management of global resources.

In addition to the overall agrobiodiversity strategy, domain-specific national programmes for genetic resources for plant, animal, forest, and aquatic genetic resources were developed. They are functional instruments to describe the detailed measures to be implemented throughout Germany in each of the domains within a certain time period, to set priorities, to facilitate the monitoring of activities and achievements, and to assist in linking the required stakeholder groups. A potential programme for microbial genetic resources is being considered. The programmes are updated from time to time to remain useful instruments for priority setting and implementation.

The implementation of the programmes is based upon the active involvement of a wide range of stakeholders and constituencies involved in all areas such as the identification, collecting, conservation, documentation, characterization, evaluation, and other uses of genetic resources for research and breeding as well as for direct uses for food and agriculture, horticulture, pomiculture, viticulture, forestry, and fisheries. The stakeholders participate in their respective fields of competence and measures based upon their existing respective responsibilities and budgets.

National expert committees for plant, animal, forest, and aquatic genetic resources, consisting of *Laender* authorities and a wide range of experts and stakeholders are in charge of guiding the implementation of the national programmes.

According to the amended scope of the genetic resources concept and the new agrobiodiversity strategy and in recognition of the usefulness of an information and coordination entity, the former Information and Coordination Centre for Genetic Resources (IGR) was renamed into Information and Coordination Centre for Biological Diversity (IBV) and was integrated in the Federal Office for Agriculture and Food (BLE) in 2005. The current tasks of the IBV relate to agrobiodiversity, in particular to genetic resources for food, agriculture, forestry, and fisheries; they include inter alia:

- To provide advice to the Federal and *Laender* ministries;
- To support the BMEL representing Germany in international bodies such as the FAO Commission on Genetic Resources (CGRFA), the European Cooperative Programme for Plant Genetic Resources (ECPGR), the European Regional Focal Point of Animal Genetic Resources (ERFP) and bodies at the European Commission;
- Provides national coordinator for ECPGR;
- Support of the development and implementation of the national programmes for plant, animal, forest and aquatic genetic resources;
- Secretariat for the national expert committees of the Federal Ministry for Agriculture (BMEL) for plant, animal, forest and aquatic genetic resources, as well as for the Scientific Advisory Board for Biodiversity and Genetic Resources;
- Data collection and documentation of national inventories as well as user-oriented central dissemination of information on occurrences, characteristics and performance of genetic resources for food, agriculture, forestry and fisheries;
- Monitoring and assessment of agrobiodiversity trends in Germany;
- Coordination of conservation activities and assistance to conservation networks;
- Facilitation of national and international support measures and programmes;
- Knowledge transfer and advisory services for political decision makers and other stakeholders;
- Biopatent monitoring and access and benefit-sharing (ABS) issues;
- Public relations and awareness raising.

The Scientific Advisory Board on Biodiversity and Genetic Resources was constituted in 2003. The Board advises the BMEL on general and fundamental issues relating to the conservation and sustainable use of biological diversity at national, EU, and international level. Members of the Board are scientists from different disciplines appointed by the Federal Ministry of Agriculture, the four chairpersons of the national expert committees on plant, animal, forest, and aquatic genetic resources, as well as the director of the IBV. The main topics to be considered by the board are:

- Biological and ecological basics;
- Economic, social and ethical evaluation;
- Development of science and technology, including genetics and breeding;
- Land use, landscaping and rural areas;
- Importance for raw materials, energy, nutrition and health;
- Promotion of strategies and concepts;
- Legal, policy and ethical issues;
- Information and communication, marketing and awareness.

While the BMEL is setting the policy framework, the implementation of the national programmes remains under the responsibility of all stakeholders. Due to the broad scope of the demanding programmes a wide range of actors are involved such as Federal and *Laender* institutes, genebanks, research institutions, fisheries, the private sector and non-governmental organizations, including universities, agricultural and horticultural actors, breeders, farmers and foresters, nature conservation, botanic and zoological gardens.

The IBV is keeping an oversight and facilitates the implementation of the national programmes through information, documentation, communication, and coordination measures. A specific website (https://genres.de/en/) is dedicated to providing the overall information platform of all programmes and stakeholders as well as the national inventories and a newsletter.

## 5. Coordination and Governance Structure of the German Plant Genetic Resources System

Given the global challenges as agreed by the 2030 Agenda for Sustainable Development with its 17 Sustainable Development Goals (SDG) and the particular importance of climate change, loss of biodiversity, and food security, the essential role of plant genetic resources for food and agriculture is evident. These resources are fundamental for further research and plant breeding to support the diversification of the agriculture and food system and contribute to the climate change adaptation processes.

**Figure 2:**
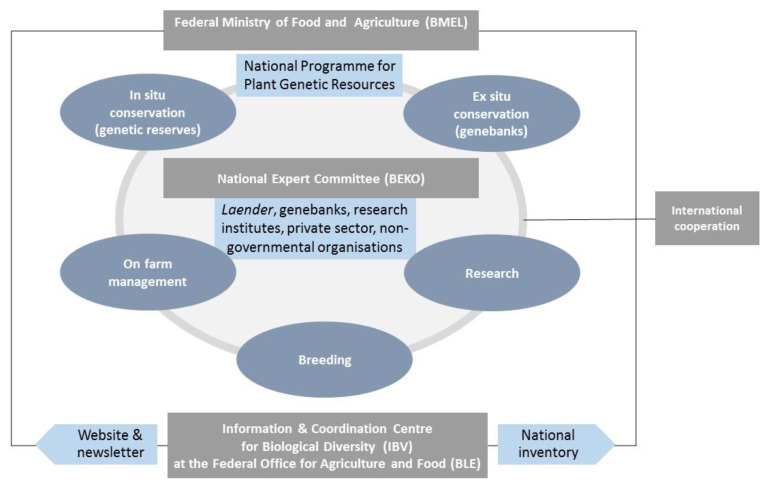
Elements of the German plant genetic resources system.

**Figure 3:**
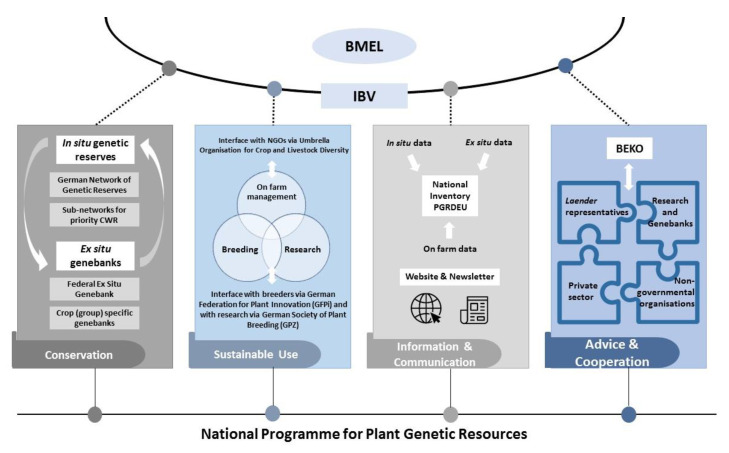
Coordination and governance structure of the German plant genetic resources system.

Based on its historical developments, the German plant genetic resources system (see Figure 2 and Figure 3) is characterized by an effective long-term conservation infrastructure with internationally recognized genebanks and well-qualified plant research institutions. A large number of breeding and seed companies operate in Germany and offer, as of July 2021, 2635 varieties of agricultural species and 640 varieties of vegetable species at the European seed catalogues to farmers. A wide range of non-governmental organizations and individuals are engaged in conservation and management of plant genetic resources on farms or in gardens.

The National Programme for the Conservation and Sustainable Use of Plant Genetic Resources of Agricultural and Horticultural Crops [19] describes the main objectives and measures to be undertaken at the national level. The first national PGR programme was adopted by the Conference of Agriculture Ministers in 2002. It was fundamentally revised in 2012. This second programme is based on the second Global Plan of Action for Plant Genetic Resources for Food and Agriculture of FAO (GPA2). It describes the political and legislative framework at national, European, and international level. The national priority activities are grouped in accordance with the main elements of GPA2, i.e., specifically address ex situ conservation, in situ conservation including both on farm management and in situ conservation of crop wild relatives (CWR), sustainable use, as well as information and documentation. The next revision and updating cycle of the national programme has already been initiated by the Ministry.

The national expert committee for PGR, called the “Advisory and Coordinating Committee for Agricultural and Horticultural Crops (BEKO)” was established by the BMEL in 2002. It consists of up to 17 members representing Federal and *Laender* authorities, professional associations and organizations from science and research, the private sector, representatives of genebanks, the in situ conservation and on farm management sector, and non-governmental organizations. The terms of office of members are five years. The BEKO has provided reports about the implementation of the national programme for the periods 2008–2014 and 2015 to 2019. Since the beginning of the current term (2020–2024), also the nature protection sector is represented through the Federal Agency for Nature Protection.

### 5.1. Ex Situ Conservation

In Germany, there are currently six national genebanks (see Table 3). These consist of more than 100 collections hosted and curated by a most varied range of actors at the Federal, *Laender,* and local level, and even by private individuals. Four of these genebanks are in fact decentralized networks that are specialized in the conservation of certain crops, namely the German Genebank for Fruit Crops, the German Genebank for Grapevine, the German Genebank for Ornamentals, and the Genebank for Crop Wild Relatives.

Despite the differences in the species conserved and the actors involved, all four decentralized genebank networks follow the same structure, which is set out in a cooperation agreement. The coordinating organizations of the two larger networks, i.e., the Genebank for Fruit Crops and the Genebank for Ornamentals, are supported by scientific advisory boards.

All genebanks conserve their accessions according to the FAO genebank standards [20]. The IPK genebank is running a quality management system since 2007 and is certified according to ISO 9001:2015. The vast majority of its collection is stored as dry seed at −18 °C. Conservation of vegetatively propagated accessions is facilitated through permanent cultivation in the field or by means of in vitro culture or cryo conservation in liquid Nitrogen.

The IBV, as a higher-level coordinating body, is a partner with defined tasks in all decentralized genebank networks. This includes the integration of the respective genebanks in the national and international processes as well as the integration of the data about the respective genebank holdings into the National Inventory of Plant Genetic Resources (PGRDEU) in Germany.

All plant genetic resources conserved in the German genebanks are distributed under the terms of the International Treaty on Plant Genetic Resources for Food and Agriculture (ITPGRFA) for the purposes of research, breeding, and training with the Standard Material Transfer Agreement (SMTA), or a special Material Transfer Agreement for ornamentals based on the SMTA. The IPK genebank provided in 2019 a total of 20,069 samples to recipients at their requests, under SMTAs; 11,351 samples were requested from abroad. The current genebank holdings are listed in Table 3.

Within the network of IBPGR base collections, the IPK held a global base collection of *Lycopersicon* and *Lupinus*, and the former FAL genebank held global collections of *Avena*, *Beta*, four *Brassica* species and *Sinapis*, as well as a European collection of *Phaseolus* [15]. Germany has placed a significant number of unique accessions within the “virtual” decentralized European collection AEGIS (A European Genebank Integrated System), which is the initiative of the European Cooperative Programme on Plant Genetic Resources (ECPGR) aiming to efficiently conserve and provide access to unique germplasm in Europe through this European Collection. The AEGIS accessions contributed by German genebanks, mostly by IPK, constitute 41% of the European collection as of July 2021. About 65% of the accessions (Table 3), specifically 75% of the IPK collection and accessions held by the CWR and fruit genebanks, are of species included in Annex I of the ITPGRFA and have been notified as part of its Multilateral System. 

### 5.2. In Situ Conservation

**Figure 4:**
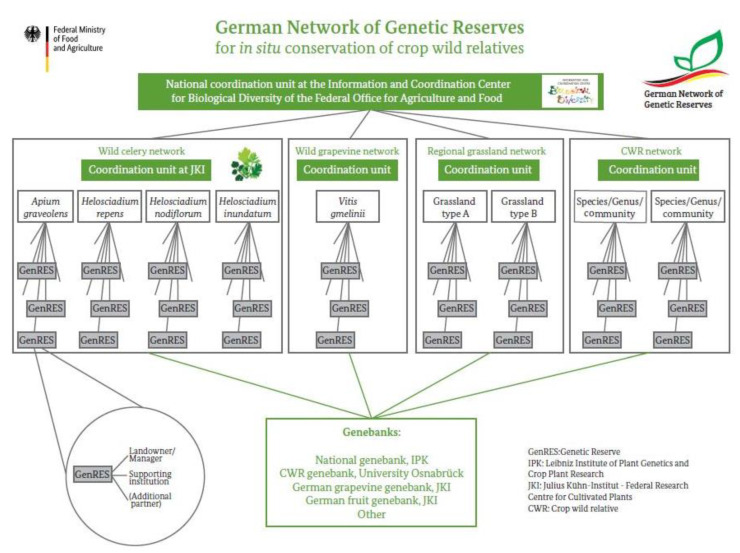
Structure of the German Network of Genetic Reserves.

Attention to in situ conservation of CWR has increased over the past decade. The “German Network of Genetic Reserves” has been established in 2019 as framework for in situ conservation of priority CWR [21]. It consists of networks for specific priority CWR. The CWR networks include genetic reserves harboring populations or plant communities identified based on agreed criteria and managed by coordination units located at agencies or institutions involved in work with PGRFA. The overall network is coordinated by the IBV (Figure 4). The German Network of Genetic Reserves has the following objectives:

- Improvement of priority CWR in situ conservation in their natural habitats, combined with complementary ex situ conservation in genebanks.
- Provision of a framework for coordination, management and integration of CWR into in situ conservation activities and for raising awareness about the importance of CWR conservation.
- Promotion of CWR utilization through documentation and the provision of freely available in situ and ex situ characterization and evaluation data in national and international information systems.
- Supporting the national PGRFA program in international cooperation and the implementation of the CBD, the GPA2, and the International Treaty on PGRFA.
- Supporting the fulfilment of international reporting obligations regarding the implementation of GPA2, the International Treaty, and the State of the World Report on PGRFA.

The first CWR specific network was the wild celery network established in 2019. Currently it is already composed of 17 wild celery genetic reserves; further reserves are in the process of being designated [22]. The coordination unit of the wild celery network is located at the Federal Research Centre for Cultivated Plants (JKI), Institute for Breeding Research on Agricultural Crops, in Quedlinburg.

In situ conservation of wild plant species is a key task of the nature protection sector, while CWR are of particular interest to the agricultural sector given their importance for plant breeding and crop improvement. Hence, their in situ conservation requires collaboration with the nature protection sector, both at the local level, when designating genetic reserves, as well as regional and federal level. This collaboration is currently being established and extended, and has *inter alia* led, as reported above, to the representation of the Federal Agency for Nature Protection in the BEKO.

### 5.3. On-Farm Sector

In Germany, there are a large number of NGOs, most of which are organized through an umbrella organization for crop and livestock diversity. This umbrella organization is also a member in the BEKO to advice on issues related to on farm conservation and management.

The EU-Regulation on Conservation Varieties adopted in 2009 created the legal prerequisite to permit and market seeds of landraces and varieties of agricultural species and vegetable species, which are relevant for the conservation of genetic resources under facilitated conditions. This was a supportive prerequisite to enable on-farm management of varieties that no longer have seed approval.

The Federal Ministry offers project funding to support on farm conservation and management, through which a large number of projects have been carried out in recent years, including some projects that investigated the recultivation of genebank accessions of old landraces from the IPK genebank.

Within the scope of the EU’s European Agricultural Fund for Rural Development (EAFRD) there is also the national cooperative funding instrument of the Federal and *Laender* governments “Improvement of the Agricultural Structure and Coastal Protection” (GAK) to support agriculture and forestry, the development of rural areas and to improve coastal and flood protection. One of the funding measures specifically serves to promote the cultivation and conservation of old landraces/varieties, that are listed on the Red List of endangered local crops in Germany. The *Laender* can offer these funding measures and receive co-financing from the Federal ministry. However, due to the priority setting by the individual *Laender*, the efforts required and the low funding volume so far only one Federal State (*Land*) offers this funding measure for plant genetic resources.

### 5.4. National Inventory 

The National Inventory of Plant Genetic Resources in Germany (PGRDEU) is the central documentation of the ex situ, in situ, and on-farm conserved plant genetic resources in Germany. This includes

- The documentation of the six national genebanks in Germany,
- The data from the “German Network of Genetic Reserves”,
- The list of priority CWRs,
- Extensive data on the historically used vegetable varieties and species from the period 1850–1950,
- The red list of endangered indigenous crop landraces/varieties in Germany,
- An inventory of on-farm actors that is currently being developed,
- Variety descriptions of genebank material from cultivation trials.

The national inventory is hosted and managed by the IBV at the BLE. It is regularly updated and, besides being a central resource for national stakeholders, it serves as data source for fulfilling international data reporting obligations to the European Catalogue of plant genetic resources EURISCO managed by the ECPGR and to FAO for SDG Indicator 2.5.1.

### 5.5. International Cooperation

The European and global collaboration in plant genetic resources for food and agriculture conservation and use is also coordinated by the PGR experts at the IBV. They coordinate the interactions with and the contributions to ECPGR. They advise and represent the Ministry in the collaboration with and sessions of the ITPGRFA and the Intergovernmental Technical Working Group for PGR of the CGRFA and take care of all international reporting obligations to the ITPGRFA, the CGRFA, and GPA. Through IBV’s various functions and roles in the BEKO and the CWR genetic reserve and genebank networks it communicates relevant international information and necessary actions to the national stakeholders and vice versa. 

## 6. Conclusions

The coordination and governance structure is functioning well since more than 20 years now. It is a light structure based on information, communication, and coordination elements but without a centralized funding structure. While this structure brought many advantages, still some challenges remain to be addressed. These issues will be elaborated based upon the plant genetic resources domain as follows.

The benefits of the structure—distributed with a central coordination—can be appreciated by the improved national cooperation with a balanced implementation of the national programme across both larger and smaller stakeholders. Additional capacities could be identified and integrated for conservation of plant genetic resources by including very small and also private actors. This is the case, for instance, in the further development of the German genebanks of fruits and ornamentals, where a number of well-qualified and motivated partners are able to contribute to the national endeavor. The comprehensive national programme, the BEKO, as well as the information and communication means allow them to participate in a fair and equitable manner.

The comprehensive representation of a wide range of stakeholders in the BEKO facilitates their engagement and contributions toward the implementation of activities in the national programme. This goes hand in hand with the official acknowledgement and recognition by reporting their valuable activities at national and international levels.

Monitoring, regular revisions, and priority setting of the national programme is facilitated by the BEKO and the supporting activities of the IBV. This approach allows to continuously integrate innovations from science, observations of the private sector, and findings of non-governmental organizations, as well as new developments from political debates and decisions, and international developments.

The capacity to collect information about activities of and contribution from numerous stakeholders, besides the well-known research institutes such as the IPK genebank in Gatersleben, allows enhanced collaboration and facilitates documentation and reporting of the German contributions toward international cooperation. Especially the ECPGR could benefit by coordinated inputs from Germany. At the same time, the active involvement of German members in ECPGR activities facilitates the harmonious implementation of the concepts developed within the ECPGR in Germany.

When developing positions for European and global processes, the German Federal Ministry of Food and Agriculture (BMEL) is benefitting from advice of the BEKO and the IBV and national programme assessments. This is especially the case for FAO processes under the CGRFA and the ITPGRFA, as well as for the CBD processes, including the Nagoya Protocol. An additional advantage of the governance structure is an improved coherence in German positions related to plant genetic resources for food and agriculture at the international level.

Besides such advantages, some challenges of this structure remain to be addressed. In particular, the information flow from *Laender* activities or non-governmental organizations related to in situ conservation and on farm activities to the IBV could be improved. IBV also would benefit from more regular information from research institutes about ongoing projects, especially those funded by third parties (e.g., the EU). Several coordination and conservation activities within the ex situ and in situ conservation networks would benefit from more long-term institutional support to stakeholders carrying out these functions. 

While policy coherence could be improved with the new governance structure for policy setting at different national agricultural for a related to plant genetic resources, processes to develop joint positions between the agricultural and environmental sectors at national and international level should still be further enhanced.

A key step in the development of the current coordination and governance structure was the need to thoroughly assess and analyze the national plant genetic resources landscape at the occasion of the German reunification. This assessment has taken into consideration the existing political and administrative structure and the distribution of competencies between the *Laender* and Federal governments. More than 30 years have passed since. Looking back today from within a stable and well-functioning plant genetic resources system, the approach to (only) centralize information and coordination functions in a permanent dedicated unit, while keeping or developing concrete implementation of conservation and use, research and breeding embedded in functioning local, regional, and federal structures or distributed networks have proven to be very sustainable and effective.

It is conducive to establishing long-term collaboration both at national and international levels, having allowed Germany to engage effectively in continued collaboration with all relevant bodies, i.e., ECPGR, FAO, ITPGRFA, and CGRFA, including respective working groups and subsidiary bodies.

This light governance structure provided by a central coordination unit, i.e., the IBV, deserves appropriately staffed offices. The scientists working at the IBV are civil servants, all experts in their respective field of genetic resources, who are entirely dedicated to carrying out the tasks listed in Section 4, for which the IBV is responsible. The financial support provided by the federal government to this permanent information and coordination unit underlines the importance, which the government does attribute to this task. It recognizes the historical developments and achievements and the difficulties faced during World War II and the post-war times. In particular, it values the fundamental importance of plant genetic resources for further research and plant breeding to support the diversification of the agricultural sector and the entire food system and to contribute to climate change adaptation processes and the protection of biological diversity.

It is recommended to establish an equivalent concept and governance structure at European level. Like Germany as a Federal state, with a multitude of stakeholders and significant differences among the *Laender*, and relevant competencies and responsibilities distributed between *Laender* and federal level, similarly Europe presents a highly diverse genetic resources landscape in terms of conservation, management, use, research and breeding, actors and (agro)ecologies, as well as relevant competencies and responsibilities. 

## Figures and Tables

**Table 1:** Size of genebank collections maintained at Gatersleben [2].

Time	Number of Accessions
1945	app. 3500
1950	12,550
1960	20,197
1970	32,489
1980	48,959
1989	65,756

**Table 2:** Most important genebank collections in Germany at IPK, FAL, and BAZ in 1992 [5].

Crop Species (Groups)	IPK	FAL ^1^	BAZ ^2^
Cereals	36.095	29.467	
Legumes	16.850	9.030	
Oil crops and fibres	5.711	3.222	
Beets and potatoes	6.580	6.265	
Fodder crops	11.142	2.797	
Tobacco	463	43	
Other agricultural crops		1.155	
Vegetables	9.962	2.237	5.000
Medicinals and spices	2.570	1.090	
Mutants etc.	2.614	1.814	900
Ornamentals	1.961		380
Fruits	1.988		163
Grapevine			2.027
Total	95.936	57.120	8.470

^1^ as of 15th August 1991, ^2^ use of estimates.

**Table 3:** Ex situ conservation of plant genetic resources in Germany (2021).

Ex Situ Conservation	Coordinating Institute	Number of Accessions ^1^	Number of Genera
Federal genebank of agricultural and horticultural crop plants	Leibniz-Institute of Plant Genetics and Crop Plant Research (IPK), Gatersleben	150,905	776
German genebank for fruit crops	Julius-Kühn-Institute (JKI), Institute for Breeding Research on fruit crops, Dresden-Pillnitz	5374	7
German genebank for grapevine	Julius-Kühn-Institute (JKI), Institute for Grapevine Breeding Geilweilerhof, Siebeldingen	4224	3
German genebank for ornamentals	Federal Plant Variety Office (BSA), Hanover	16,016	75
German genebank for crop wild relatives	Botanic Garden, University Osnabrueck	4711	178
German genebank for tobacco	NiCoTa GmbH, Rheinstetten	788	1
Total number of accessions		182,018	

^1^ Source: PGRDEU, 21 July 2021.

## Data Availability

Publicly available datasets were analyzed in this study. This data can be found here: https://pgrdeu.genres.de/ex-situ-bestaende/suche-nach-genbanken/?L=0.
